# Microbial diversity of camel milk production environments in Xinjiang, China: a full-length 16S rRNA survey of farm environmental matrices across Altay and Hami regions

**DOI:** 10.3389/fmicb.2026.1937458

**Published:** 2026-09-15

**Authors:** Yating Wu, Shuai Wang, Xianlan Ma, Xiaoxiao Lou, Nan Zheng, Wei Shao, Lu Meng, He Chen, Cheng Wang, Yankun Zhao

**Affiliations:** 1 Ministry of Agriculture and Rural Affairs-Laboratory of Quality and Safety Risk Assessment for Agro-Products, Agricultural Product Quality and Safety Risk Assessment Laboratory, Institute of Quality Standards and Testing Technology for Agro-Products, Xinjiang Academy of Agricultural Sciences, Urumqi, China; 2 Xinjiang Meat and Milk Herbivore Nutrition Laboratory, College of Animal Science, Xinjiang Agricultural University, Urumqi, China; 3 Key Laboratory for Quality and Safety Control for Milk and Dairy Products of Ministry of Agriculture and Rural Affairs, Institute of Animal Science, Chinese Academy of Agricultural Science, Beijing, China

**Keywords:** microbial diversity, production environment, raw camel milk, single-molecule real-time sequencing, source tracking

## Abstract

**Introduction:**

Camel milk production in Xinjiang, China is predominantly managed under extensive pastoral systems, yet the taxonomic and functional profiles of key production-associated environmental matrices remain poorly characterized.

**Methods:**

This study employed PacBio Single-Molecule Real-Time (SMRT) full-length 16S rRNA gene sequencing to characterize microbial communities across five environmental matrices (bedding, feces, teat skin, feed, and soil) from camel breeding farms in the Altay and Hami regions of Xinjiang.

**Results:**

After removing chloroplast and mitochondrial sequences, the dominant phyla across all samples were *Firmicutes* (43.20%), *Proteobacteria* (20.12%), and *Actinobacteriota* (12.02%). Alpha diversity analysis revealed significantly higher richness in fecal and feed microbial communities in Altay compared to Hami (Padj < 0.05). Beta diversity analyses (PCoA, NMDS, PERMANOVA) confirmed distinct taxonomic profiles between the two regions, driven by both regional and sample-type effects. Functional profiling indicated conserved metabolic potentials across regions, with enrichment of pathways related to amino acid transport and energy conversion.

**Discussion:**

This study establishes a foundational baseline of the camel farm environmental microbiome in Xinjiang, informing targeted hygiene interventions and future studies investigating environmental contributions to raw camel milk quality. A key limitation is the absence of raw milk samples, precluding direct inference of microbial transmission routes to milk.

## 1 Introduction

Camel milk production in Xinjiang, China, is primarily conducted in extensive or semi-extensive pastoral systems, where lactating camels are housed in open-air pens with natural bedding (straw or native grasses) and fed a mixed diet of locally grown alfalfa and native forage species (e.g., Haloxylon ammodendron in Altay, *Capparis spinosa* in Hami). Milking is typically performed twice daily in open-air parlors, with minimal pre-milking teat disinfection—a common practice in smallholder systems. Key environmental habitats potentially contributing to raw milk microbiota include: direct-contact reservoirs (teat apex epidermis, teat orifice); indirect-contact reservoirs (pen bedding, feeding troughs, soil surrounding the parlor); and fecal material, which is a major source of environmental inoculation due to free-ranging grazing (Ferrer et al., 2013; Martin et al., 2021). These habitats are characterized by distinct physicochemical properties: teat skin is a mesophilic, lipid-rich interface; bedding and soil are variable in moisture and organic matter content; and feed is plant-derived with high cellulose levels. Understanding habitat-specific microbial ecology is critical to optimize hygiene practices tailored to camel production systems.

It is critical to distinguish between “microbial transfer”—the natural inoculation of raw milk with taxa from environmental or host-associated reservoirs—and “microbial transfer,” which implies the introduction of spoilage or pathogenic taxa at levels exceeding safety thresholds. The former is a universal feature of raw milk production, while the latter is context-dependent and requires quantitative assessment of viable cell loads and virulence potential. Recent work by Alvanou et al. (2024) in small ruminants demonstrated that habitat-specific microbiota (e.g., teat skin, bedding) are major determinants of raw milk taxonomic composition, with regional biogeography explaining 32% of variation in milk microbiome structure. This framework provides a critical foundation for tracing environmental contributions to camel milk microbiota, which remains understudied.

Raw milk harbors a complex and dynamic microbiome, thriving in a nutrient-rich aqueous environment that supports rapid microbial proliferation. These microbial consortia encompass spoilage agents, pathogens, beneficial probiotics, and technologically relevant starter cultures (Wang et al., 2019; Gosselin et al., 2019; Moineau-Jean et al., 2019; Celano et al., 2022). Following milking and during refrigerated storage, microbial dynamics persist, ultimately influencing the microbiological quality of milk upon arrival at processing facilities (Castilho et al., 2018; Isele et al., 2019; Srisengfa et al., 2016). The raw milk microbiota is typically characterized by Gram-positive taxa, including *Bacillus*, *Micrococcus*, and *Staphylococcus*, alongside various lactic acid bacteria (LAB), such as *Lactococcus* and *Streptococcus* (Peruzi et al., 2019). Conversely, Gram-negative psychrotrophs, particularly *Pseudomonas*, *Aeromonas*, and *Enterobacteriaceae* members (e.g., *Enterobacter*, *Hafnia*, *Klebsiella*), pose significant spoilage risks due to their resilience. Yeasts, including *Candida* and *Kazachstania*, also contribute to the fungal diversity of raw milk (Krewinkel et al., 2015).

Psychrotrophic bacteria, such as *Pseudomonas* spp., *Aeromonas* spp., and *Bacillus* spp., dominate the cold-chain ecology (Qin et al., 2014). Of particular concern are the thermostable enzymes, specifically lipases and proteases, secreted by these microorganisms. These enzymes can withstand pasteurization and ultra-high temperature (UHT) treatments, representing the primary agents of spoilage that compromise the sensory and functional quality of dairy products. Specifically, lipases catalyze triglyceride hydrolysis, resulting in lipolytic rancidity and butyric acid off-flavors (Jia et al., 2021). Characterizing the microbial ecology of production-associated environments provides foundational data to inform targeted hygiene interventions, as these matrices represent potential reservoirs of taxa relevant to milk quality (Xiang et al., 2024).

The taxonomic composition of raw milk is primarily dictated by direct-contact microbial reservoirs, notably the teat apex, udder skin, and milking apparatus. Secondary influences stem from indirect-contact environments, including bedding materials, feces, feed, water sources, and the broader barn and parlor environments (Stoeckel et al., 2016). Subsequent microbial transfer can occur during milking, transportation, and storage, posing substantial risks to both the safety and processing quality of the final product. Consequently, characterizing the microbial ecology of the production environment is critical for ensuring product safety and optimizing downstream processing protocols.

Traditional culture-independent methods, such as Single-Molecule Real-Time (SMRT) sequencing, bypass the limitations of microbial cultivation by analyzing total DNA extracted directly from samples. This technology offers superior resolution and throughput compared to conventional culture-dependent microbiological techniques (Fenelon et al., 2020). By applying SMRT sequencing to metagenomic DNA, researchers can achieve precise taxonomic classification down to the species level, significantly accelerating the diagnostic timeline (Hou et al., 2016). Furthermore, third-generation sequencing facilitates a holistic interrogation of microbial genomic architectures within environmental niches, thereby maximizing the exploitable potential of bioresources (Zhou et al., 2024). Specifically, targeting the full-length 16S rRNA gene allows for high-fidelity resolution of bacterial taxa, enabling localization to the species level (Liang et al., 2022).

Herein, we conducted a comprehensive investigation into the microbial ecology of five environmental matrices associated with camel milk production (feces, bedding, feed, teat skin, and soil) to elucidate the overall microbial diversity. The primary objectives were to characterize the taxonomic and functional profiles of these key environmental reservoirs, to inform hygiene management, disease prevention, and potential beneficial microbial resource discovery. This work establishes a theoretical framework for understanding the dynamics of microbial transfer within camel milk production systems in Xinjiang, China. By mitigating potential microbial transfer risks and deepening insights into production environment microbiology, this study furnishes detailed contextual data to support dairy processing, enhance safety assurance, and facilitate novel strain discovery. Ultimately, these findings lay a robust scientific foundation for the systematic monitoring of raw milk quality and safety pending validation with paired raw milk samples.

## 2 Materials and methods

### 2.1 Sample collection

Sampling was conducted across 6 commercial camel breeding farms (3 farms in Altay Prefecture: 47°52′N, 88°08′E; 3 farms in Hami City: 42°49′N, 93°31′E) during the summer season of 2023 (July to September). For each region, 5 sample types were collected: (1) Bedding: ~50 g of surface bedding material (top 5 cm) from 3 independent pens per farm; (2) Feed: ~50 g of fresh roughage (mixed alfalfa and local forage) from feeding troughs in 3 independent pens per farm; (3) Feces: fresh fecal samples collected directly from the rectum of 3 lactating camels per farm using sterile spatulas; (4) Soil: ~50 g of surface soil (top 5 cm) from 3 locations within 5 m of each farm’s milking parlor; (5) Teat skin: swabs collected from the distal teat apex (2 cm^2^ area surrounding the teat orifice) of 3 lactating camels per farm using sterile polyester-tipped swabs (Copan, Italy) moistened with sterile phosphate-buffered saline (PBS, pH 7.4). All animal sampling was performed before routine pre-milking teat cleaning. Three biological replicates per sample type per region were defined as independent samples collected from distinct farms (i.e., one sample per farm, resulting in three independent biological replicates per category). Samples were not pooled at the farm level. A total of 30 samples were retained after quality control, coded as”07”(Altay) or”09”(Hami) with suffixes-1 to-3 indicating independent replicates (full sample metadata provided in Supplementary Table S1). All samples were placed in sterile, sealed containers, transported to the laboratory on ice within 4 h of collection, and stored at −80 °C until DNA extraction (maximum storage time: 14 days). To prevent cross-microbial transfer, sterile gloves were changed between sampling individual animals, and all sampling tools were flame-sterilized or replaced between samples. Negative control samples (sterile PBS for swabs, sterile empty containers for solid matrices) were processed in parallel during sampling, transport, and laboratory processing to monitor for background microbial transfer.

### 2.2 DNA extraction

Genomic DNA was extracted from all samples using the TIANamp Bacterial DNA Kit (Tiangen Biotech, Beijing, China), following the manufacturer’s protocol. The concentration and purity (A260/A280 ratio) of the extracted DNA were quantified using a NanoPhotometer® N60 (Implen GmbH, Munich, Germany). Subsequently, DNA integrity and fragment size were verified by electrophoresis on a 1% (w/v) agarose gel. Only samples with high purity (OD260/280 ratio between 1.8 and 2.0) and intact high-molecular-weight bands were selected for subsequent library construction.

### 2.3 PCR amplification

The full-length 16S rRNA gene (V1-V9 region) was amplified using barcoded primers 27F (5′-AGRGTTYGATYMTGGCTCAG-3′) and 1492R (5′-RGYTACCTTGTTACGACTT-3′). PCR reactions were performed in 25 μL volumes containing 12.5 μL 2 × TransStart Fastpfu PCR Mix (TransGen Biotech), 1 μL each of forward and reverse primers (10 μM), 2 μL template DNA (10 ng/μL), and 8.5 μL sterile nuclease-free water. Cycling conditions were: 95 °C for 3 min; 25 cycles of 95 °C for 30 s, 55 °C for 30 s, 72 °C for 90 s; final extension at 72 °C for 10 min. A preliminary trial on 6 representative samples confirmed 25 cycles as the minimum required to obtain sufficient amplicon yield while minimizing PCR bias. Each sample was amplified in triplicate; replicate amplicons were pooled and visualized on 2% agarose gels. Target bands (~1,450 bp) were excised and purified using the AxyPrep DNA Gel Recovery Kit (Axygen Biosciences), eluted in Tris–HCl buffer (pH 8.8), and quantified using the QuantiFluor™ ST Blue Fluorescence System (Promega).

### 2.4 Fluorescence quantification

Library concentrations were accurately determined using the QuantiFluor™ ST Blue Fluorescence System (Promega, Madison, WI, USA). Subsequently, libraries were normalized and pooled in equimolar ratios based on the sequencing platform’s requirements.

### 2.5 Library construction and sequencing

SMRTbell libraries were constructed using the SMRTbell Express Template Prep Kit 2.0 (Pacific Biosciences, Menlo Park, CA, USA) following the manufacturer’s instructions. Briefly, the PCR amplicons were purified using AMPure PB beads (Pacific Biosciences) to remove short fragments and primers. Size selection was performed via 2% agarose gel electrophoresis to ensure the integrity of the inserts. The purified DNA was eluted in TE buffer and quantified using a Qubit dsDNA HS Assay Kit (Invitrogen, Carlsbad, CA, USA). Finally, the double-stranded DNA templates were denatured and bound to the polymerase to form sequencing-ready complexes for Single-Molecule Real-Time (SMRT) sequencing.

### 2.6 SMRT sequencing

Sequencing was performed on a PacBio Sequel II system (Pacific Biosciences, Menlo Park, CA, USA) following the manufacturer’s guidelines. The sequencing process relies on Single-Molecule Real-Time (SMRT) technology, wherein DNA polymerase complexes are immobilized at the bottom of zero-mode waveguides (ZMWs). Sequencing primers and the SMRTbell templates were bound to form sequencing-ready complexes. Upon loading into the SMRT Cell, the diffusion of nucleotides allowed for real-time monitoring of fluorescence pulses as phospholinked nucleotides were incorporated. This setup enabled the generation of high-fidelity reads, facilitating comprehensive profiling of the microbial communities.

### 2.7 Bioinformatics analysis

Raw subreads from PacBio Sequel II sequencing were processed using the ccs v6.4.0 algorithm with parameters: --min-passes 3, --min-rq 0.99, to generate HiFi reads. Quality filtering was performed using cutadapt v4.4 with parameters: -q 20, --length 1,000, to remove low-quality bases and short reads. Primer trimming was conducted using cutadapt with exact matching to remove 27F/1492R primer sequences. Chimeric sequences were detected and removed using VSEARCH v2.23.0 with the --uchime_ref command against the SILVA 138 reference database. Denoising and Amplicon Sequence Variant (ASV) generation were performed using the QIIME2 (v2023.2) plugin “q2-dada2” (dada2 v1.28.0) with default parameters. Taxonomic assignment was conducted using the QIIME2 plugin “q2-feature-classifier” with a naive Bayes classifier trained on the SILVA 138 16S rRNA reference database, using a confidence threshold of 0.7. Chloroplast and mitochondrial sequences were identified via SILVA 138 annotation. The relative abundance of these organelle-derived sequences was quantified for all samples and is reported separately in the Results section as a descriptive metric of sample composition. For all downstream ecological and statistical analyses—including alpha diversity, beta diversity (Bray–Curtis dissimilarity-based PCoA, NMDS, PERMANOVA), LEfSe biomarker discovery, and COG functional prediction—chloroplast and mitochondrial ASVs were excluded from the dataset. This two-stage approach ensures transparency in sample composition while avoiding bias from eukaryotic 16S signals in microbial ecology analyses. Rarefaction was performed at a uniform depth of 5,000 reads per sample (the minimum retained read count across all samples); a total of 1,247 high-quality ASVs were retained after filtering low-abundance features (<0.001% relative abundance) and low-prevalence features (present in <2 samples). A negative control (PCR-grade water) processed in parallel yielded 12 reads (0.008% of the total dataset), confirming negligible reagent or cross-contamination. Alpha diversity metrics [Chao1, ACE, Shannon, inverse Simpson (1/D)] and beta diversity (Bray–Curtis dissimilarity) were calculated using QIIME2 native plugins. Alpha diversity metrics [Chao1, ACE, Shannon, Simpson Dominance (D)] and beta diversity (Bray–Curtis dissimilarity) were calculated using QIIME2 native plugins. For all statistical comparisons, Farm ID was included as a random blocking factor in PERMANOVA models (Adonis2, strata = farm_id) to restrict permutations within farms. All downstream analyses were performed at the ASV level; ASV-based clustering was not employed in this study.

## 3 Results

### 3.1 Overall microbiome analysis

Taxonomic profiling revealed a total of 35 bacterial phyla and 100 genera across all samples in the filtered dataset (after removal of chloroplast and mitochondrial sequences). The majority of bacterial ASVs were assigned to three predominant phyla: *Firmicutes* (43.20%), *Proteobacteria* (20.12%), and *Actinobacteriota* (12.02%). Unfiltered feed samples contained high proportions of chloroplast (54.47%) and mitochondrial (17.08%) sequences, reflecting plant-derived material and eukaryotic cells—a common occurrence in 16S rRNA amplicon sequencing of forage samples. These organelle-derived sequences were excluded from all subsequent ecological analyses. It is noteworthy that the high abundance of chloroplast and mitochondrial sequences likely reflects the presence of plant-derived material and eukaryotic cells in the samples, which is a common occurrence in 16S rRNA amplicon sequencing of environmental samples (Figure 1).

**Figure 1:**
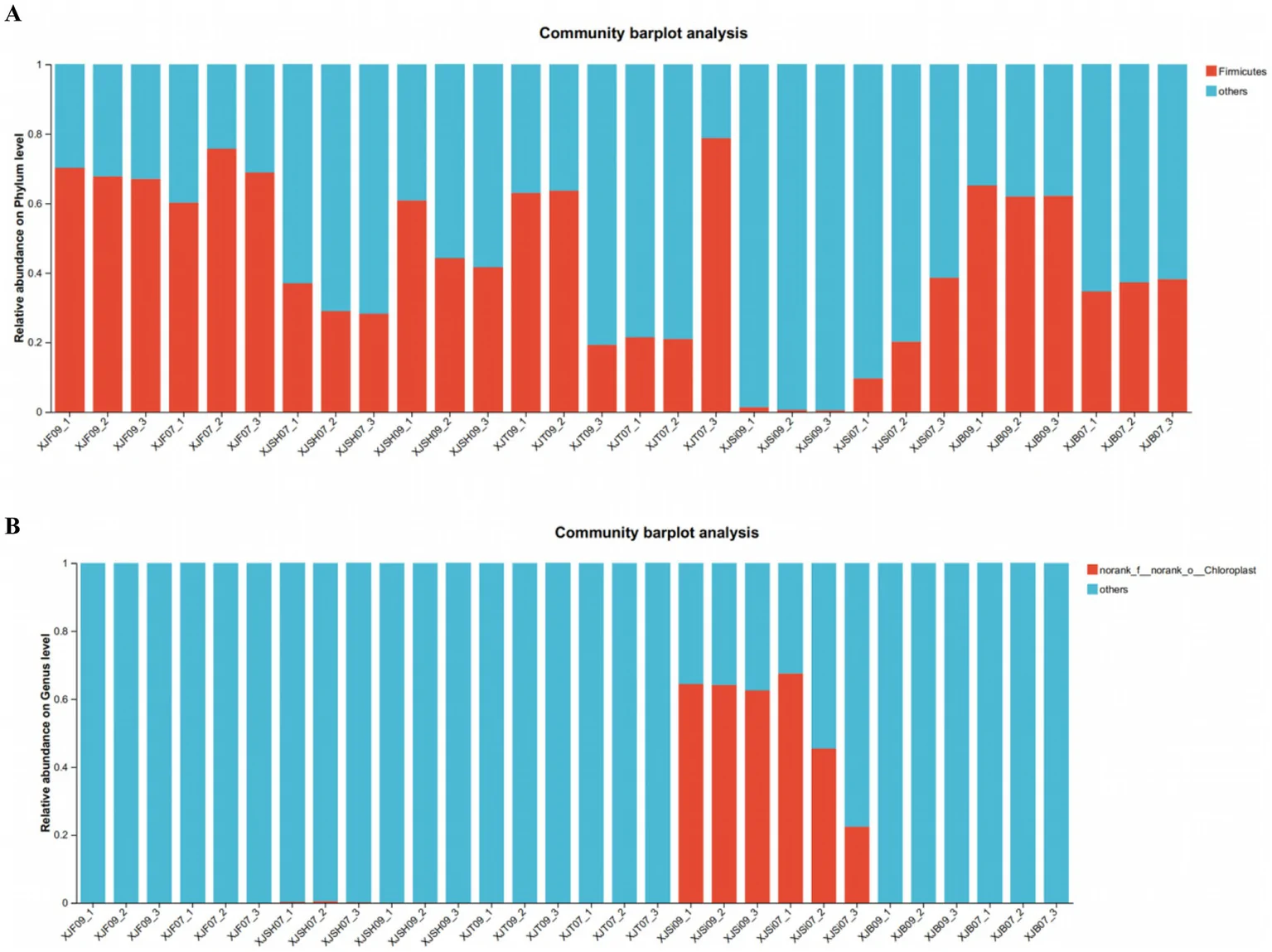
Relative abundance of phylum **(A)** and genus in samples **(B)**.

### 3.2 Microbiota diversity based on alpha and beta analysis

Alpha diversity was evaluated using the Chao1 and ACE indices (richness), alongside the Shannon (diversity) and inverse Simpson (1/D, evenness) indices. Two-way ANOVA with Tukey’s *post-hoc* correction was used to test for differences between regions, sample types, and their interaction; adjusted *p*-values (Padj) are reported for all comparisons. The ACE index revealed significantly higher community richness in fecal samples from Altay (332.33 ± 30.15) compared to Hami (239.07 ± 6.61; Padj = 0.012, effect size = 1.24, 95% CI [32.7, 153.8]), consistent with higher richness in Altay feed samples (348.24 ± 44.89 vs. 294.00 ± 17.09 in Hami; Padj = 0.041, effect size = 0.98, 95% CI [2.1, 106.4]). No significant regional differences were observed in soil richness (ACE: 323.33 ± 74.77 in Altay vs. 294.00 ± 17.09 in Hami; Padj = 0.312). Chao1 values followed a similar pattern, with significantly higher fecal richness in Altay (327.47 ± 31.62 vs. 235.50 ± 5.88 in Hami; Padj = 0.008, effect size = 1.37, 95% CI [41.2, 142.7]). No significant regional differences were observed in Shannon or inverse Simpson indices across most sample types (all Padj > 0.05), indicating comparable diversity and evenness between the two regions (Table 1).

**Table 1:** Alpha-diversity indices of bacterial communities in environmental samples from two regions.

Sample	Chao 1 index	Ace index	Shannon index	Simpson index
XJB07	335.49 ± 45.28	342.85 ± 49.18	3.91 ± 0.12	0.04 ± 0.00
XJB09	350.54 ± 43.96	348.24 ± 44.89	3.77 ± 0.24	0.05 ± 0.02
XJF07	327.47 ± 31.62a	348.24 ± 44.89	3.94 ± 0.10	0.04 ± 0.01
XJF09	235.50 ± 5.88a	294.00 ± 17.09a	3.65 ± 0.18	0.04 ± 0.01
XJSH07	333.67 ± 76.74	323.33 ± 74.77	3.14 ± 0.42	0.15 ± 0.07
XJSH09	309.48 ± 21.66	294.00 ± 17.09	3.43 ± 0.19	0.07 ± 0.02
XJSI07	335.77 ± 64.94	334.67 ± 65.12	2.87 ± 0.56	0.03 ± 0.01
XJSI09	340.50 ± 55.38	345.78 ± 56.90	3.52 ± 0.32	0.06 ± 0.02
XJT07	225.77 ± 45.39	230.11 ± 46.75	2.72 ± 0.71	0.18 ± 0.12
XJT09	250.33 ± 29.99	255.67 ± 30.83	2.89 ± 0.51	0.14 ± 0.08

Different superscript letters indicate significant differences between regions (two-way ANOVA, Tukey’s *post-hoc* test, Padj < 0.05). Simpson index values represent Simpson Dominance (D), where lower values indicate greater diversity and evenness.

To visualize structural variations in microbial composition, three ordination methods were applied to Bray–Curtis dissimilarity matrices: (1) Principal Coordinates Analysis (PCoA); (2) Non-Metric Multidimensional Scaling (NMDS); and (3) Principal Component Analysis (PCA, applied to Hellinger-transformed abundance data, Figure 2A). The PCoA plot (Figure 2B) revealed distinct clustering patterns between the Altay and Hami groups. ANOSIM (999 permutations) confirmed that inter-group differences were significantly larger than intra-group variations (*R* = 0.7503, *p* = 0.001). NMDS ordination yielded a low stress value of 0.094 (Figure 2C), indicating excellent fit of the data in reduced dimensional space. To partition variation in microbial composition, a factorial PERMANOVA was performed on Bray–Curtis dissimilarity matrices with region, sample type, and their interaction as fixed effects (999 permutations). Region (Pseudo-*F* = 18.72, *p* = 0.001), sample type (Pseudo-*F* = 32.45, *p* = 0.001), and their interaction (Pseudo-*F* = 4.12, *p* = 0.003) all significantly explained variation in community structure. Homogeneity of multivariate dispersion was assessed using the betadisper function in the vegan R package; no significant differences in dispersion were observed between regions (*F* = 1.23, *p* = 0.281) or sample types (*F* = 0.98, *p* = 0.432), confirming that PERMANOVA results are not biased by unequal group variances.

**Figure 2:**
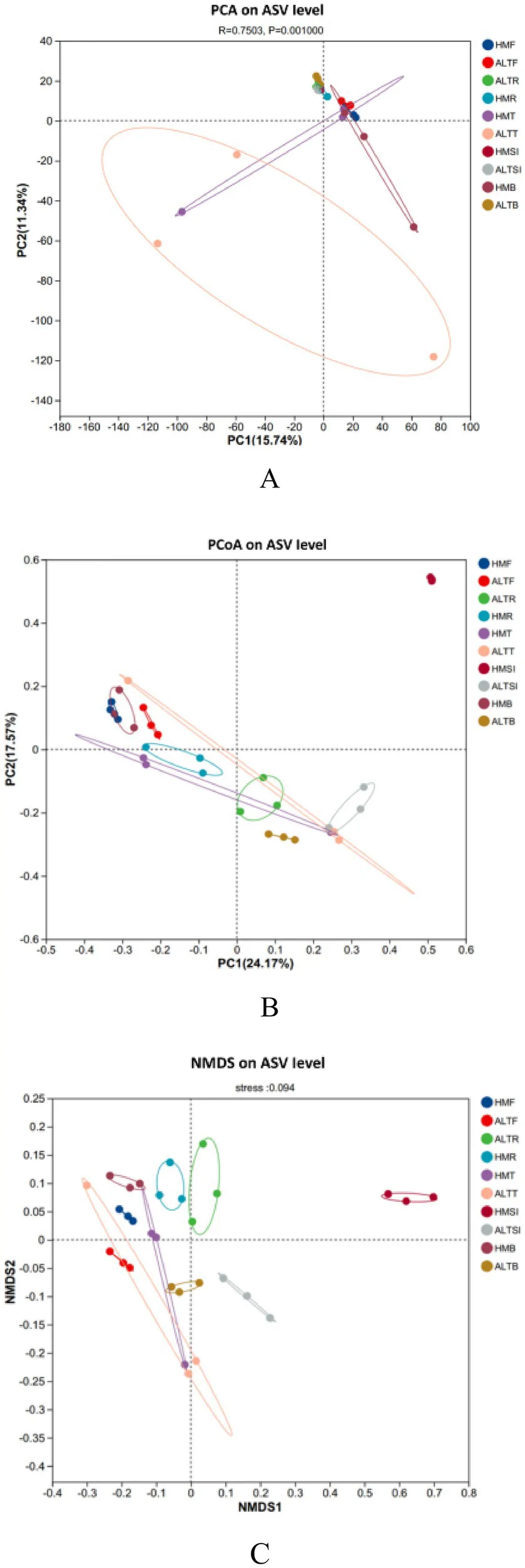
Principal component analysis based on the Euclidean distance algorithm [PCA, **(A)**], principal coordinate analysis [PCoA, **(B)**], and non-metric multidimensional scaling analysis [NMDS, **(C)**].

The heatmap visualization of genus-level abundance (Figure 3) revealed distinct clustering patterns between the Altay and Hami regions, reflecting significant differences in microbial composition. Achromobacter was consistently detected across teat skin samples from both regions. In contrast, sequences identified as mitochondria (likely representing eukaryotic contaminants or degraded host DNA) were exclusively found in feed samples from Hami.

**Figure 3:**
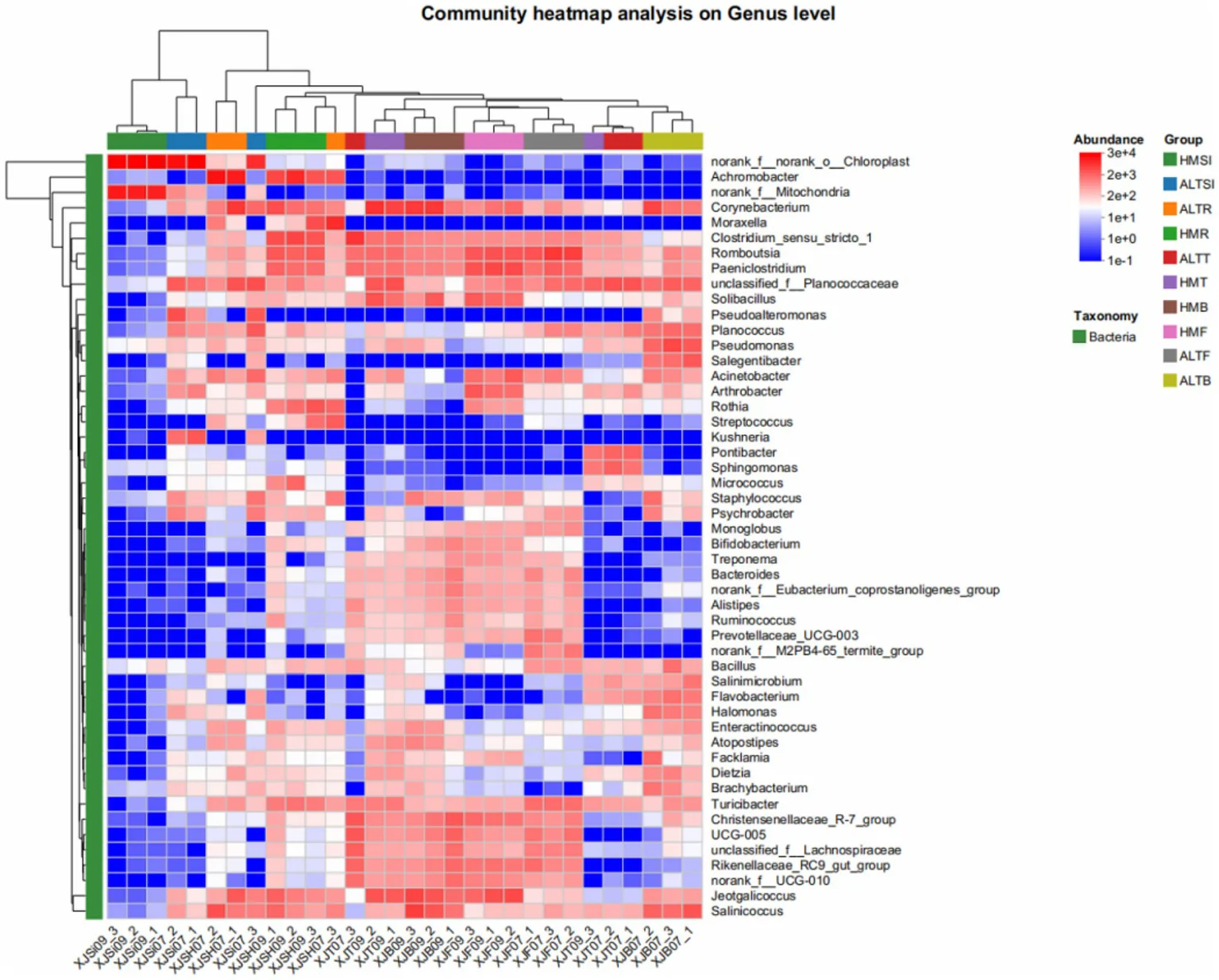
Heatmap of genus-level abundance clustering across all samples. Only bacterial ASVs (post-removal of chloroplast and mitochondrial sequences) are included.

Anaerobic taxa, including *Clostridium* sensu stricto, *Romboutsia*, and *Paeniclostridium*, exhibited a broad distribution across soil and fecal samples in both regions, with an extended presence in feed and bedding samples specifically within the Hami region. Conversely, specific genera, such as *Bifidobacterium*, *Treponema*, and *Bacteroides*, were predominantly restricted to soil, bedding, and fecal samples in the Hami region, with sporadic detection in Altay feces.

Certain taxa demonstrated regional specificity; for instance, *Bacillus* was exclusively identified in fecal, soil, and bedding samples from the Altay region. Similarly, *Salinimicrobium*, *Flavobacterium*, and *Halomonas* were unique to the soil and bedding of Altay. In Hami, *Christensenellaceae* R-7 group and *Rikenellaceae* RC9 group were prevalent in soil and bedding. The genus *Jeotgalicoccus* was primarily associated with teat skin and bedding in Hami, whereas *Salinicoccus* showed a ubiquitous presence across bedding and skin in both regions. These distinct distribution profiles underscore the feasibility of differentiating bacterial communities between the two regions based on environmental reservoirs.

### 3.3 Common and difference analysis of bacterial compositions

Taxonomic composition analysis revealed distinct dominant profiles across the five environmental matrices (Figure 4). Fecal samples were dominated by *Romboutsia* (10.77%), followed by *Paeniclostridium* (8.10%) and *Jeotgalicoccus* (4.85%) (Figure 4A). Teat skin samples exhibited a distinct profile, with *Achromobacter* spp. (22.23%) and *Moraxella* (6.29%) being predominant, alongside *Clostridium* sensu stricto (5.00%) (Figure 4B). Feed samples were characterized by members of the *Planococcaceae* family (3.63%) in the filtered dataset (post-organelle removal). The high abundance of chloroplast (54.47%) and mitochondrial (17.08%) sequences in unfiltered feed samples reflects the plant-based composition of camel roughage and is reported separately as a descriptive metric, not included in ecological analyses (Figure 4C). Soil samples were primarily composed of *Planococcaceae* (7.05%), *Clostridium sensu stricto* (4.16%), and *Romboutsia ilealis* (2.95%) (Figure 4D). Finally, bedding samples were dominated by *Corynebacterium maris* (5.98%), *Jeotgalicoccus* spp. (5.61%), and *Salinicoccus* spp. (4.62%) (Figure 4E).

**Figure 4:**
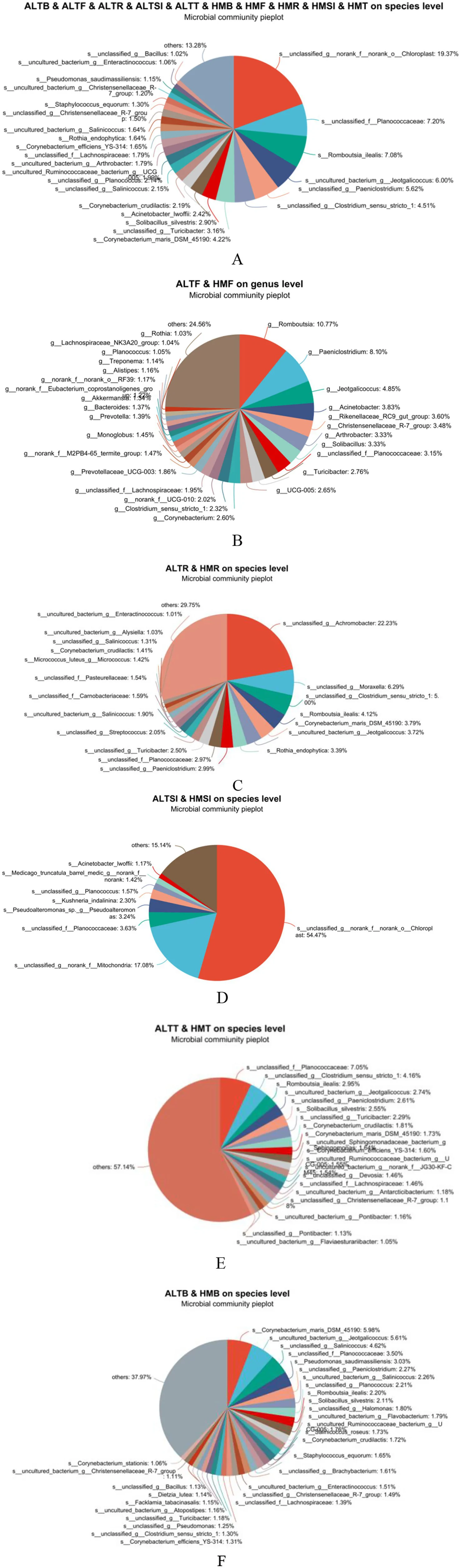
Pie charts illustrating the relative abundance of the top 10 bacterial genera in each sample type from Altay (07) and Hami (09) regions. **(A)** Fecal samples; **(B)** Teat skin samples; **(C)** Feed samples; **(D)** Soil samples; **(E)** Bedding samples. Only the filtered dataset (post-removal of chloroplast and mitochondrial sequences) was used for taxonomic profiling of bacterial genera. **(F)** Pie chart depicting microbial community at species level for ALTB and HMB groups.

To identify the specific bacterial taxa driving significant differences in community composition between the Altay and Hami regions, biomarker analysis was performed using LEfSe: alpha value for Kruskal-Wallis test = 0.05, LDA score threshold = 4.0, strict effect size filtering. Groups for LEfSe analysis were defined as (1) region (Altay vs. Hami) within each sample type to identify sample-type-specific biomarkers; and (2) sample type within each region to identify environment-specific biomarkers. Biomarker discovery was performed using LEfSe (v1.0) on the filtered dataset (post-removal of chloroplast and mitochondrial sequences). Taxa labeled as “uncultured,” “unranked,” or “unclassified” were excluded from the dataset prior to analysis.

As illustrated in Figures 5A–C, *Firmicutes* was the dominant phylum in fecal samples from Altay, whereas *Actinobacteriota* predominated in those from Hami. A similar pattern was observed in teat skin samples (*Firmicutes* in Altay vs. *Actinobacteriota* in Hami). In soil samples, *Actinobacteriota* dominated in Altay, while *Firmicutes* was predominant in Hami. Feed samples lacked a dominant phylum in Altay but were dominated by *Firmicutes* in Hami. Additionally, bedding samples in Altay were characterized by *Actinobacteriota*, whereas those in Hami were dominated by *Firmicutes.*

**Figure 5:**
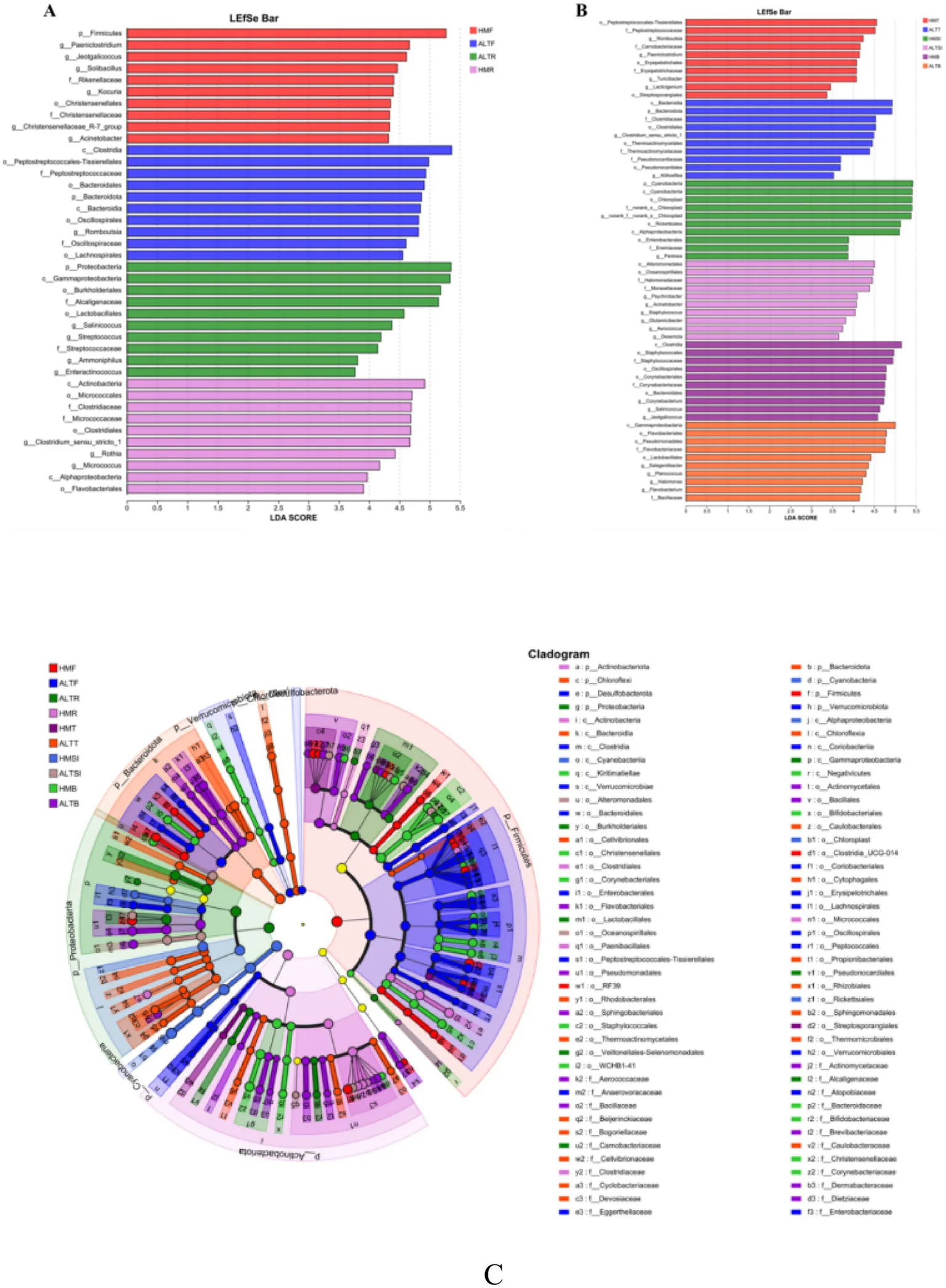
Linear discriminant analysis (LDA) effect size (LEfSe) method **(A)**; Developmental dendrogram of samples from two regions representing significant differences with an LDA threshold of 4.0 LDA discriminant histograms of genera of different abundance between the two regions **(B,C)**.

These findings are consistent with the overall beta-diversity trends observed in previous sections. Furthermore, LEfSe identified 10 bacterial phyla with LDA scores exceeding 4.0, highlighting distinct taxonomic signatures between the regions.

### 3.4 Model prediction and predictive functions

Functional profiling based on the Clusters of Orthologous Groups (COG) database revealed that the majority of genes were annotated to metabolic functions, including global and overview maps, as well as secondary metabolite biosynthesis (Figure 6A). Predominant pathways specifically encompassed amino acid transport and metabolism, alongside the transport and metabolism of inorganic ions. Furthermore, secondary metabolite biosynthesis was linked to energy production and conversion, as well as translation, ribosomal structure, and biogenesis.

**Figure 6:**
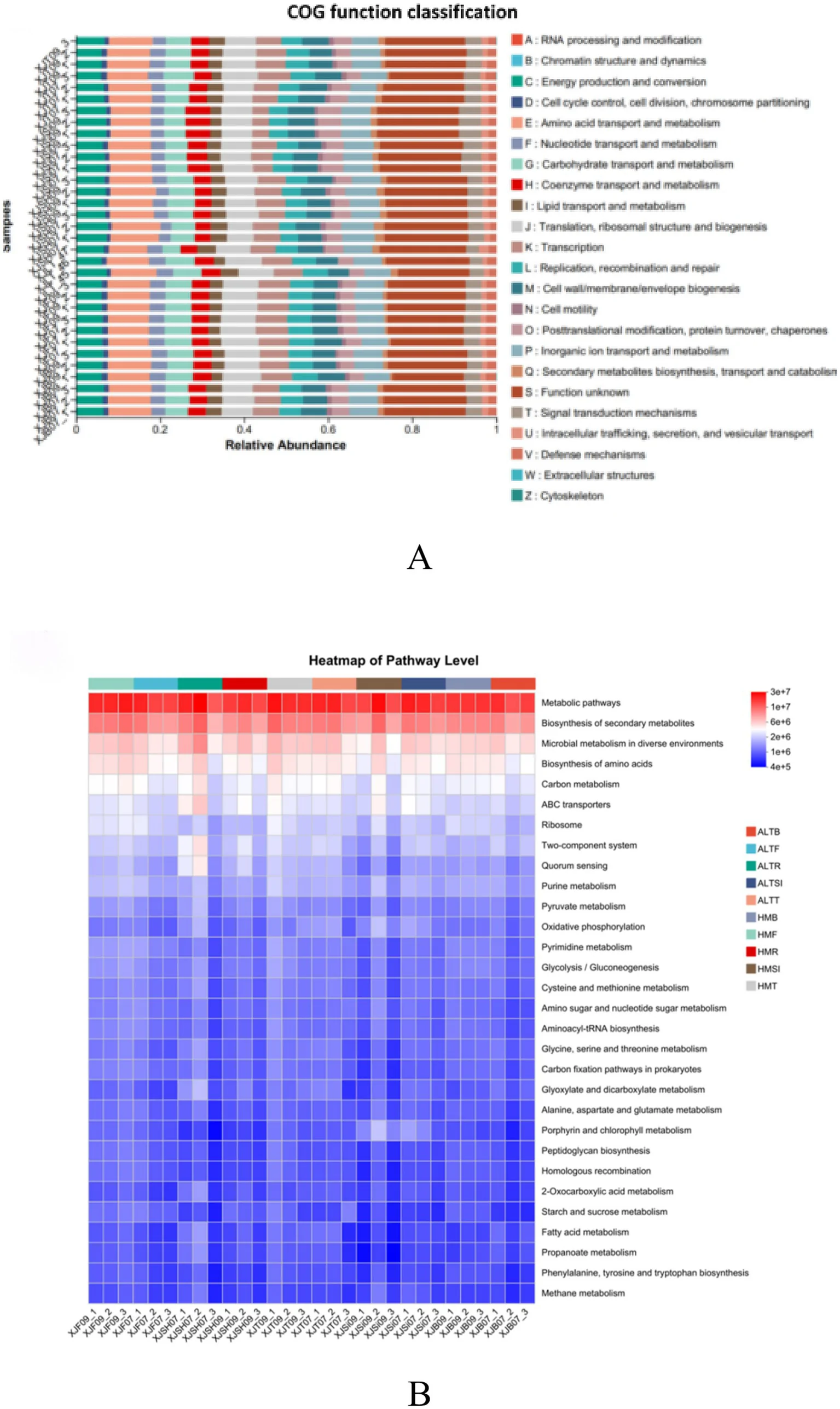
Predictive functional analysis of microbial communities based on the COG database. **(A)** Similar COG functional classification profiles across all samples; **(B)** Key metabolic pathways associated with COG-encoded proteins and the structure of the overall cellular metabolic network.

These results highlight the interconnected nature of the cellular metabolic network, emphasizing systemic functionality over isolated biochemical reactions (Figure 6B). Comparative analysis indicated highly consistent COG functional profiles across both regions, suggesting conserved core metabolic potentials despite variations in taxonomic composition. It is important to note that functional predictions from 16S rRNA gene data are inferential and may not fully represent the actual functional capacity of the microbial community.

## 4 Discussion

This study provides a comprehensive characterization of the microbial ecology across five key environmental matrices associated with camel milk production in the Altay and Hami regions of Xinjiang. The dominant phyla identified—*Firmicutes* (43.20%), *Proteobacteria* (20.12%), and *Actinobacteriota* (12.02%)—differ significantly from those reported in pasteurized milk studies. For instance, Ding et al. (2020) observed that *Proteobacteria* and *Firmicutes* each exceeded 50% in HTST-treated milk, with *Pseudomonas* dominating the genera. The taxonomic profiles observed here differ from those reported in pasteurized camel milk studies, though direct comparisons are limited by differences in sample type (environmental vs. milk), animal species, geographical origin, and sequencing methodologies (Ferrer et al., 2013). Pasteurization-induced shifts in milk microbiota cannot be inferred from the current environmental dataset.

When compared to other non-bovine milks, our results align with the trends reported by Luoyizha et al. (2020) for donkey milk, particularly regarding the abundance of *Bacteroidota* (~10%) (Palii et al., 2020). However, unlike Zhang et al. (2023), who found *Firmicutes* to constitute 96% of mare’s milk, our camel milk samples exhibited a more balanced distribution among the top phyla. This variation likely stems from species-specific factors, regional climates, and dietary differences. Similarly, Ouyang et al. (2022) reported *Proteobacteria* dominance (43.3–84.0%) in seawater, noting *Cyanobacteria* (0.3–10.4%); our observed *Cyanobacteria* (7.91%) falls within this range, although in the context of ruminant feces/feed, these signals likely represent plant-derived chloroplast sequences rather than free-living cyanobacteria (Sánchez-Baracaldo, 2025).

Environmental sourcing analysis revealed distinct distribution patterns. In Altay, Firmicutes was highly prevalent in teat skin (49.58%) and soil (48.65%), suggesting these as potential reservoirs of taxa relevant to milk quality (Martin et al., 2021). Conversely, in Hami, fecal samples showed high abundances of *Cyanobacteria* (68.34%) and *Firmicutes* (29.07%). These findings corroborate Dong et al. (2021), who identified feces as a critical vector for Firmicutes transmission to milking equipment and storage tanks in dairy systems, though direct transmission to camel milk was not assessed in this study. Furthermore, Zhang et al. (2023) highlighted that *Proteobacteria* often originate from raw milk and water sources, which aligns with our observation of high *Proteobacteria* in Hami feed (21.80%) and soil (20.46%). The high prevalence of Cyanobacteria (often indicative of chloroplast sequences from plant matter) in feces and feed highlights the influence of roughage composition on the microbial landscape.

Functional profiling based on the COG database indicated that the microbiome is heavily geared toward metabolic activities, including amino acid transport and energy conversion. The consistent functional profiles across both regions, despite taxonomic variations, suggest a degree of functional redundancy within these ecosystems. Notably, the enrichment of *Firmicutes* and *Bacteroidota* is relevant to the production of short-chain fatty acids (SCFAs) like butyrate and propionate, which can suppress pro-inflammatory cytokines and strengthen epithelial barriers (Zhi et al., 2022). The detection of taxa previously reported as psychrotrophic in dairy environments (e.g., *Pseudomonas*, *Bacillus*) suggests a potential risk of spoilage-associated enzyme production, though this study did not measure viable cell counts, enzyme activity, or spoilage phenotypes, and thus cannot confirm functional relevance.(Xiang et al., 2024; Fanelli et al., 2020).

Ensuring raw milk safety is paramount for public health and industry sustainability. Microbial transfer of raw milk is often driven by *Pseudomonas* spp. and other psychrotrophs, though the current study does not assess direct microbial transfer risks. The observed taxonomic distributions highlight environmental matrices that warrant prioritized monitoring in camel dairy farms, supporting the utility of integrating environmental surveillance with traditional milk quality testing. By mapping the environmental sources of key taxa, this work provides actionable evidence for upgrading quality surveillance systems and optimizing production practices in Xinjiang’s dairy sector (Pierezan et al., 2022). Ultimately, the integration of SMRT sequencing data with traditional microbiological parameters offers a robust scientific foundation for regulatory bodies to implement precision monitoring across the entire dairy supply chain (Yuan et al., 2022), pending validation with paired raw milk samples.

A limitation of this study is the absence of raw milk samples; thus, all inferences regarding potential contributions of environmental taxa to milk microbiota are speculative. A key limitation of 16S rRNA amplicon sequencing is the inability to distinguish between viable, viable-but-nonculturable, and dead cells; thus, the presence of a given taxon does not confirm its metabolic activity or pathogenic potential. Future studies incorporating raw milk specimens and quantitative source-tracking approaches (e.g., SourceTracker2 or FEAST) are needed to validate the transmission routes inferred from this environmental survey.

A key priority for future camel dairy research is to validate whether the habitat-specific microbial signatures identified here are transferred to raw milk, as demonstrated in small ruminants by Alvanou et al. (2024). The current environmental dataset provides a critical baseline for such paired studies, which would enable: (1) quantitative source tracking of milk microbiota to specific habitats (e.g., teat skin vs. bedding); (2) validation of regional biomarkers for geographic origin authentication of camel milk; and (3) targeted hygiene interventions to reduce transfer of spoilage/pathogenic taxa. Additionally, the inability of 16S rRNA sequencing to distinguish viable vs. non-viable cells remains a limitation—future work should integrate propidium monoazide (PMA) treatment to quantify viable microbiota in environmental reservoirs (Peruzi et al., 2019).

## 5 Conclusion

This study systematically mapped the microbial landscapes of five key environmental matrices associated with camel milk production in Xinjiang’s Altay and Hami regions. We characterized the taxonomic structure of these environmental reservoirs and assessed their potential implications for raw milk safety and processing stability, though direct transmission to milk was not evaluated. The observed regional differences in taxonomic composition and conserved functional profiles provide a baseline for environmental monitoring in camel dairy systems. However, without direct analysis of raw milk samples, the conclusions regarding microbial transmission into milk remain preliminary and require validation in future studies. These findings provide a scientific foundation for improving hygiene protocols and quality surveillance in camel dairy production systems.

## Data Availability

The dataset has been published and is publicly accessible at: https://data.mendeley.com/datasets/7h6m79dc22/1.

## References

[ref1] Alvanou M. V. Loukovitis D. Melfou K. Giantsis I. A. (2024). Utility of dairy microbiome as a tool for authentication and traceability. Open Life Sci. 19:20220983. doi: 10.1515/biol-2022-0983, 39479351 PMC11524395

[ref2] Castilho N. P. A. Colombo M. Todorov S. D. Nero L. A. (2018). Beneficial properties of lactic acid bacteria naturally present in dairy production. BMC Microbiol. 18:219. doi: 10.1186/s12866-018-1356-8, 30567551 PMC6300030

[ref3] Celano G. Costantino G. Vacca M. D’Auria M. Calabrese F. M. De Angelis M. . (2022). Effect of seasonality on microbiological variability of raw cow milk from Apulian dairy farms in Italy. Microbiol. Spectrum 10:e0051422. doi: 10.1128/spectrum.00514-22, 35972127 PMC9602280

[ref4] Ding R. Liu Y. Yue X. Wang Y. Zhang Y. Chen Q. (2020). High-throughput sequencing provides new insights into the roles and implications of core microbiota present in pasteurized milk. Food Res. Int. 137:109586. doi: 10.1016/j.foodres.2020.109586, 33233194

[ref5] Dong L. Pang X. Tang S. Wang J. Zhang Y. (2021). High-throughput sequencing reveals bacterial diversity in raw milk production environment and production chain in Tangshan city of China. Food Sci. Anim. Resour. 41, 452–467. doi: 10.5851/kosfa.2021.e10, 34017954 PMC8112312

[ref7] Fanelli F. Cho G. S. Böhnlein C. Kabisch J. Franz C. M. A. P. (2020). Microbial quality and safety of milk and milk products in the 21st century. Compr. Rev. Food Sci. Food Saf. 19, 2013–2049. doi: 10.1111/1541-4337.12568, 33337106

[ref8] Fenelon M. A. Gleeson D. Hill C. Cotter P. D. Guinane C. M. (2020). Tracking the dairy microbiota from farm bulk tank to skimmed milk powder. mSystems. 5:e00226-20. 10.1128/mSystems.00226-20 PMC7141888 32265313

[ref9] Ferrer M. Lanza F. Bargiela R. Almansa C. Puchades-Carrasco L. Moya A. (2013). Microbiota from the distal guts of lean and obese adolescents exhibit partial functional redundancy besides clear differences in community structure. Environ. Microbiol. 15, 211–226. doi: 10.1111/j.1462-2920.2012.02845.x, 22891823

[ref6] Gosselin V. Dufour S. Adkins P. R. F. Middleton J. R. Barkema H. W. (2019). Persistence of coagulase-negative staphylococcal intramammary infections in dairy goats. J. Dairy Res. 86, 211–216. 10.1017/S0022029919000311 31138341

[ref10] Hou Q. Bian Y. Zhang W. Liu X. Huang L. Deng Z. (2016). Using PacBio long-read high-throughput microbial gene amplicon sequencing to evaluate infant formula safety. J. Agric. Food Chem. 64, 6993–7001. 10.1021/acs.jafc.6b01817 27500310

[ref11] Isele D. Hahne J. Berning J. Baur C. Krewinkel M. von Neubeck M. . (2019). The contribution of fast growing, psychrotrophic microorganisms on biodiversity of refrigerated raw cow's milk with high bacterial counts and their food spoilage potential. Food Microbiol. 79, 11–19. 10.1016/j.fm.2018.10.019 30621865

[ref12] Jia Z. Huang L. Wei Z. Wang X. (2021). Dynamic kinetic analysis of growth of *Listeria monocytogenes* in pasteurized cow milk. J. Dairy Sci. 104, 2654–2667. doi: 10.3168/jds.2020-19442, 33455764

[ref13] Krewinkel M. Kranz B. von Neubeck M. Wenning M. Scherer S. (2015). Biodiversity of refrigerated raw milk microbiota and their enzymatic spoilage potential. Int. J. Food Microbiol. 211, 57–65. doi: 10.1016/j.ijfoodmicro.2015.07.001, 26173200

[ref14] Liang L. He L. Qu T. Wang J. (2022). Single-molecule real-time sequencing reveals differences in bacterial diversity in raw milk in different regions and seasons in China. J. Dairy Sci. 105, 5669–5684. doi: 10.3168/jds.2021-21445, 35599031

[ref15] Luoyizha W. Wu X. Zhang M. Wang J. (2020). Compared analysis of microbial diversity in donkey milk from Xinjiang and Shandong of China through high-throughput sequencing. Food Res. Int. 137:109684. doi: 10.1016/j.foodres.2020.109684, 33233260

[ref16] Martin N. H. Wiedmann M. Torres-Frenzel P. (2021). Invited review: controlling dairy product spoilage to reduce food loss and waste. J. Dairy Sci. 104, 1251–1261. doi: 10.3168/jds.2020-19130, 33309352

[ref17] Moineau-Jean A. Champagne C. P. Roy D. Raymond Y. LaPointe G. (2019). Effect of Greek-style yogurt manufacturing processes on starter and probiotic bacteria populations during storage. Int. Dairy J. 93, 35–44. doi: 10.1016/j.idairyj.2019.02.003

[ref18] Ouyang L. Chen X. Zhang W. Wang J. (2022). High throughput sequencing reveals distinct bacterial communities and functional diversity in two typical coastal bays. J. Mar. Sci. Eng. 10:1878. doi: 10.3390/jmse10121878

[ref19] Palii A. P. Paliy A. P. Rodionova K. O. (2020). Microbial contamination of cow’s milk and operator hygiene. Ukr. J. Ecol. 10, 392–397.

[ref20] Peruzi G. A. S. Bruzaroski S. R. Conti A. C. M. Nero L. A. (2019). Short communication: effect of bactofugation of raw milk on counts and microbial diversity of psychrotrophs. J. Dairy Sci. 102, 7794–7799. 10.3168/jds.2018-16148 31279557

[ref21] Pierezan M. D. Maran B. M. Maran E. M. Nero L. A. (2022). Relevant safety aspects of raw milk for dairy foods processing. Adv. Food Nutr. Res. 100, 211–264. doi: 10.1016/bs.afnr.2022.01.001, 35659353

[ref22] Qin X. Cheng J. Qiu Y. Wang J. (2014). Characterization of psychrotrophic and thermoduric bacteria in raw milk using a multi-omics approach. Microb. Genom. 10:1311. 10.1099/mgen.0.001311 PMC11540130 39504117

[ref23] Sánchez-Baracaldo P. (2025). Blue-green algae (cyanobacteria). Nat. Ecol. Evol. 9:2174. doi: 10.1038/s41559-025-02858-6, 40957957

[ref24] Srisengfa Y. Laird M. McLeod J. Griffiths M. W. (2016). The core and seasonal microbiota of raw bovine milk in tanker trucks and the impact of transfer to a milk processing facility. MBio 7:e00836-16. doi: 10.1128/mBio.00836-16, 27555305 PMC4999540

[ref25] Stoeckel M. Lidolt M. Achberger V. Hinrichs J. (2016). Growth of *Pseudomonas weihenstephanensis*, *Pseudomonas proteolytica* and *Pseudomonas* sp. in raw milk: impact of residual heat-stable enzyme activity on stability of UHT milk during shelf-life. Int. Dairy J. 59, 20–28. doi: 10.1016/j.idairyj.2016.02.045

[ref26] Wang N. I. Yuan L. Sadiq F. A. He G. (2019). Insights into psychrotrophic bacteria in raw milk: a review. J. Food Prot. 82, 1148–1159. doi: 10.4315/0362-028X.JFP-19-032, 31225978

[ref27] Xiang Q. Xia Y. Fang S. Wang J. (2024). Enzymatic debittering of cheese flavoring and bitterness characterization of peptide mixture using sensory and peptidomics approach. Food Chem. 440:138229. doi: 10.1016/j.foodchem.2023.138229, 38159315

[ref28] Yuan H. Han S. Zhang Y. Wang J. (2022). Microbial properties of raw milk throughout the year and their relationships to quality parameters. Foods 11:3077. doi: 10.3390/foods11193077, 36230153 PMC9563975

[ref29] Zhang X. Chang L. Zhang J. Wang J. (2023). 16S rDNA high throughput sequencing bacterial diversity analysis of cow mastitis. Indian J. Anim. Res. 57, 1578–1583.

[ref30] Zhi W. Chen R. Tang K. Wang J. (2022). Research on the gut microbiota of Hainan black goat. Animals 12:3129. doi: 10.3390/ani12223129, 36428357 PMC9686789

[ref31] Zhou X. Chen L. Song Z. Wang J. (2024). Pathogenic bacteria and fungi in bioaerosols from specialized hospitals in Shandong province, East China. Environ. Pollut. 341:122922. 10.1016/j.envpol.2023.122922 37984476

