# Supplementary material for: Microbial diversity of camel milk production environments in Xinjiang, China: a full-length 16S rRNA survey of farm environmental matrices across Altay and Hami regions
Source: Front Microbiol. 2026 Sep 15;17:1937458. doi: 10.3389/fmicb.2026.1937458 (PMC13619886; doi:10.3389/fmicb.2026.1937458)
Supplement: Supplementary file 1 [file Table_1.DOCX]

Supplementary Material

# 1 Supplementary Tables

Table_S1_Sample_List

| Sample ID | Region | Sample Type |
| --- | --- | --- |
| XJB07-1 | Altay | Bedding |
| XJB07-2 | Altay | Bedding |
| XJB07-3 | Altay | Bedding |
| XJF07-1 | Altay | Feces |
| XJF07-2 | Altay | Feces |
| XJF07-3 | Altay | Feces |
| XJSH07-1 | Altay | Teat Skin |
| XJSH07-2 | Altay | Teat Skin |
| XJSH07-3 | Altay | Teat Skin |
| XJSI07-1 | Altay | Feed |
| XJSI07-2 | Altay | Feed |
| XJSI07-3 | Altay | Feed |
| XJT07-1 | Altay | Soil |
| XJT07-2 | Altay | Soil |
| XJT07-3 | Altay | Soil |
| XJB09-1 | Hami | Bedding |
| XJB09-2 | Hami | Bedding |
| XJB09-3 | Hami | Bedding |
| XJF09-1 | Hami | Feces |
| XJF09-2 | Hami | Feces |
| XJF09-3 | Hami | Feces |
| XJSH09-1 | Hami | Teat Skin |
| XJSH09-2 | Hami | Teat Skin |
| XJSH09-3 | Hami | Teat Skin |
| XJSI09-1 | Hami | Feed |
| XJSI09-2 | Hami | Feed |
| XJSI09-3 | Hami | Feed |
| XJT09-1 | Hami | Soil |
| XJT09-2 | Hami | Soil |
| XJT09-3 | Hami | Soil |

The following abbreviations are used in this manuscript:

| ASV | Amplicon Sequence Variant |
| --- | --- |
| COG | Clusters of Orthologous Groups |
| HTST | High-Temperature Short-Time |
| LAB | Lactic Acid Bacteria |
| LEfSe | Linear Discriminant Analysis Effect Size |
| NMDS | Non-metric Multidimensional Scaling |
| PCoA | Principal Coordinates Analysis |
| SMRT | Single-Molecule Real-Time (sequencing) |
| UHT | Ultra-High Temperature |
